# Enhancing of chemical compound and drug name recognition using representative tag scheme and fine-grained tokenization

**DOI:** 10.1186/1758-2946-7-S1-S14

**Published:** 2015-01-19

**Authors:** Hong-Jie Dai, Po-Ting Lai, Yung-Chun Chang, Richard Tzong-Han Tsai

**Affiliations:** 1 Graduate Institute of Biomedical Informatics, College of Medical Science and Technology, Taipei Medical University, Taipei, Taiwan; 2 Institute of Information Science, Academia Sinica, Taipei, Taiwan; 3 Department of Information Management, National Taiwan University, Taipei, Taiwan; 4 Department of Computer Science and Information Engineering, National Central University, Taoyuan, Taiwan

## Abstract

**Background:**

The functions of chemical compounds and drugs that affect biological processes and their particular effect on the onset and treatment of diseases have attracted increasing interest with the advancement of research in the life sciences. To extract knowledge from the extensive literatures on such compounds and drugs, the organizers of BioCreative IV administered the CHEMical Compound and Drug Named Entity Recognition (CHEMDNER) task to establish a standard dataset for evaluating state-of-the-art chemical entity recognition methods.

**Methods:**

This study introduces the approach of our CHEMDNER system. Instead of emphasizing the development of novel feature sets for machine learning, this study investigates the effect of various tag schemes on the recognition of the names of chemicals and drugs by using conditional random fields. Experiments were conducted using combinations of different tokenization strategies and tag schemes to investigate the effects of tag set selection and tokenization method on the CHEMDNER task.

**Results:**

This study presents the performance of CHEMDNER of three more representative tag schemes-IOBE, IOBES, and IOB_12_E-when applied to a widely utilized IOB tag set and combined with the coarse-/fine-grained tokenization methods. The experimental results thus reveal that the fine-grained tokenization strategy performance best in terms of precision, recall and F-scores when the IOBES tag set was utilized. The IOBES model with fine-grained tokenization yielded the best-F-scores in the six chemical entity categories other than the "Multiple" entity category. Nonetheless, no significant improvement was observed when a more representative tag schemes was used with the coarse or fine-grained tokenization rules. The best F-scores that were achieved using the developed system on the test dataset of the CHEMDNER task were 0.833 and 0.815 for the chemical documents indexing and the chemical entity mention recognition tasks, respectively.

**Conclusions:**

The results herein highlight the importance of tag set selection and the use of different tokenization strategies. Fine-grained tokenization combined with the tag set IOBES most effectively recognizes chemical and drug names. To the best of the authors' knowledge, this investigation is the first comprehensive investigation use of various tag set schemes combined with different tokenization strategies for the recognition of chemical entities.

## Background

Studies on the effects of chemical and drug on organismal growth and development under various conditions are very valuable. As a result, both the academia and industry are interesting in finding new ways to retrieve and access chemical compound and drug-related information from narrative texts in a manner that minimizes the required effort. RI Dogan, GC Murray, A Névéol and Z Lu [1] established that apart from bibliographic queries (such as author name and article title), chemical entities are some of the terms frequently used to browse and search the PubMed database. As research within the biomedical field has evolved, advancements of experimental techniques, the accumulation of experiences and the ease of access to publications around the world have all contributed to the acceleration of biomedical studies, generating enormous repositories of scientific journals and papers. Hence, traditional manual methods of identifying chemical entities in articles and associating them to databases are no longer suffice to meet the needs of researchers, motivating the development of several chemical entity recognition approaches that are based on natural language processing approaches [2,3]. In contrast to previously proposed gene mention recognition and normalization task [4,5], the recognition of chemical entities has yet to been much improved using limited standard corpus and evaluation tools. For example, P Corbett and A Copestake [6] evaluated OSCAR3 using a corpus consisting of 500 PubMed abstracts. Unfortunately, that corpus remains unavailable to the public. To accelerate the research into CHEMical Compound and Drug Name Entity Recognition (CHEMDNER), a CHEMDNER task was set by BioCreative IV [7] to improve the efficiency and accuracy of chemical and drug recognition, to the benefit of both academia and industry.

Identifying chemical entities in text is hindered by the existence of highly varied ways of naming them. Such names include trivial or brand names (such as Tylenol), systematic International Union of Pure and Applied Chemistry (IUPAC) names such as 6-keto prostaglandin F(1α), generic or family names (such as alcohols), company codes (such as ICI204636), molecular formulas (such as H_2_SO_4_) and identifiers associated with various databases (such as CHEBI:28262). Additionally, many of these names are used abbreviated (such as to DMS for dimethyl sulfate). Although nomenclature organizations such as IUPAC have been striving for systematic naming in the biochemical field, most of their rules are treated only as suggestions rather than regulations, leaving ample room for variation in their use.

As indicated in the overview paper of the BioCreative CHEMDNER task [7], the majority of the approaches that were used by participating teams to detect chemical entities were the machine learning method based on conditional random fields (CRFs), used with a variety of feature sets, along with chemistry-related lexical resources and several pre-/post-processing rules. Despite the promising results of the BioCreative CHEMDNER task, most effort has been applied to the development of various feature sets. The tag set has received much less attention. Accordingly, this study focuses on various tag sets and their effect on the performance of chemical entity recognition with CRFs. Experiments were performed using combinations of different tokenization strategies and tag schemes to elucidate the effects of tag set selection and tokenization strategy on the identification of chemical and drug entities. The results thus demonstrate that tag set selection is as important as feature selection.

Chemical entities can be classified into various categories [3]. For instance, based on the annotation guideline for the CHEMDNER task, the sentence,

"Different samples will be collected and analyzed for five PCAHs including pyrene, benzo(a)anthracene, benzo(e)pyrene, benzoflouroanthene, and benzo(a)pyrene."

includes two types of chemical entity, and should be annotated as follows.

"Different samples will be collected and analyzed for five [**PCAHs **_ABBREVIATION_] including [**pyrene**_SYSTEMATIC_], [**benzo(a)anthracene**_SYSTEMATIC_], [**benzo(e)pyrene**_SYSTEMATIC_], [**benzoflouroanthene**_SYSTEMATIC_], and [**benzo(a)pyrene**_SYSTEMATIC_]."

ABBREVIATION indicates that "PCAHs" is an acronym for a chemical compound. SYSTEMATIC indicates that "pyrene", "benzo(a)anthracene", "benzo(e)pyrene", "benzoflouroanthene", and "benzo(a)pyrene" are IUPAC names. The recognition of chemicals under different categories can facilitate the following chemical entity normalization system to link the mentions to their corresponding database records. For example, the abbreviated name "PCAHs" is linked to "polycyclic aromatic hydrocarbons", and the systematic name "pyrene" is linked to the ChEBI ID: 39106. Therefore, this study not only presents the unified results concerning the combinations of various tag schemes and tokenization strategies obtained using the official CHEMDNER evaluation script, but also present results for each of the seven categories of chemical names that were defined in the CHEMDNER task. The influence of the proposed tag set on the recognition performance of each individual category is also examined.

## Methods

In this study, the CHEMDNER task is formulated as a sequence labelling problem. The same feature sets that are utilized for machine learning are developed with various tag sets and their effect on the recognition of chemicals is studied. The subsequent sections firstly detail the proposed tag sets and the employed machine learning model. Then, the workflow of the proposed system and the feature sets that are used in it are elucidated.

### Conditional random fields

**Figure 1:**
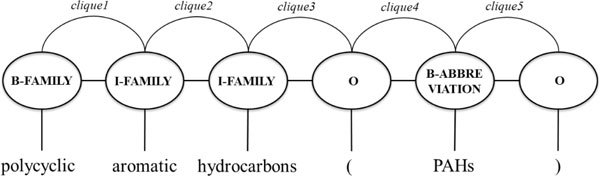
**Graphical representation of "polycyclic aromatic hydrocarbons (PAHs)" tagged as "[B-FAMILY, I-FAMILY, I-FAMILY, O, B-ABBREVIATION, O]"**.

The machine learning model that is utilized herein is CRFs model [8]. CRFs are undirected graphical models that are trained to maximize a conditional probability of random variables, and have been successfully used in numerous sequential labelling tasks, such as named entity recognition and Chinese word segmentation. In this study, the linear-chain CRF was employed to recognize sequentially the boundaries of chemical entities moving from the first token to the final token of a tokenized sentence. Given an input sequence of tokens *W*, a linear-chain CRF computes the probability associated with its corresponding hidden labelled sequence *Y *as

$${\text{p}}_{\lambda }\left(Y\text{|}W\right)=\frac{1}{Z\left(W\right)}exp\left(\underset{c\in C}{\sum }\underset{i}{\sum }\lambda_{i}f_{i}\left(y_{c-1},y_{c},W,c\right)\right)$$

where *Z*(*W*) is the normalization factor that makes the probability of all state sequences sum to one; *C *is the set of all cliques in this sentence, and *c *is a single clique, which reflects the position of the current word, as displayed in Figure 1. The function *f_i_*(*y*_*c*-1_, *y_c_*, *W*, *c*) is a binary-valued feature function whose weight is *λ_i_*. Large positive values of *λ_i _*indicate a preference for such the corresponding feature. The Feature set subsection will describe the feature functions that are utilized in this study.

### Tag set selection

The IOB scheme is the most tag scheme used for establishing the tag set in the biomedical named entity recognition task. For example, the state-of-the-art system for recognizing mentions of genes [9] adopts the IOB tag set in its bi-directional parsing algorithm. Even in the CHEMDNER task, the top-ranked systems [10,11] used the IOB scheme. Figure 1 presents the graphical representation in CRF of "polycyclic aromatic hydrocarbons (PAHs)" tagged using the IOB tag set (B-FAMILY, I-FAMILY, I-FAMILY, O, B-ABBREVIATION, O). The scheme suggests a model to learn and identify the **B**eginning, the **I**nside and the **O**utside of a particular category of chemical entities.

Various tag schemes for the task of Chinese word segmentation have been proposed and showed a promising improvement. For instance, N Xue [12] proposed the use of a new tag to represent a Chinese "word" if it forms only a word by itself. H Zhao, C-N Huang, M Li and B-L Lu [13] concentrated on the subdivision of the beginning of Chinese words into tags like B_1 _and B_2 _to better capture longer words. Unlike in the Chinese word segmentation task, in the CHEMDNER task, category information of chemicals is associated with the tag set, leading to a high computational cost and training time. Accordingly, the IOB scheme is delicately extended into four different schemes, whose relative performances when applied to the CHEMDNER task were compared. In particular, motivated by the works on Chinese word segmentation, the tags E and S, which stand for "**E**nd of the entity" and "**S**ingle-word entity", are added to the IOB tag set to form a four-(IOBE) and five-(IOBES) tag sets. Accordingly, the labelling sequence in Figure 1 becomes B-FAMILY, I-FAMILY, E-FAMILY, O, S-ABBREVIATION, and O when the five tag set is used. In the experiments, the B tag is also split into B1 and B2 tags to form another five-tag scheme, IOB_12_E. These extended schemes provide more precise machine learning material and establish more intelligent models.

### System workflow

**Figure 2:**
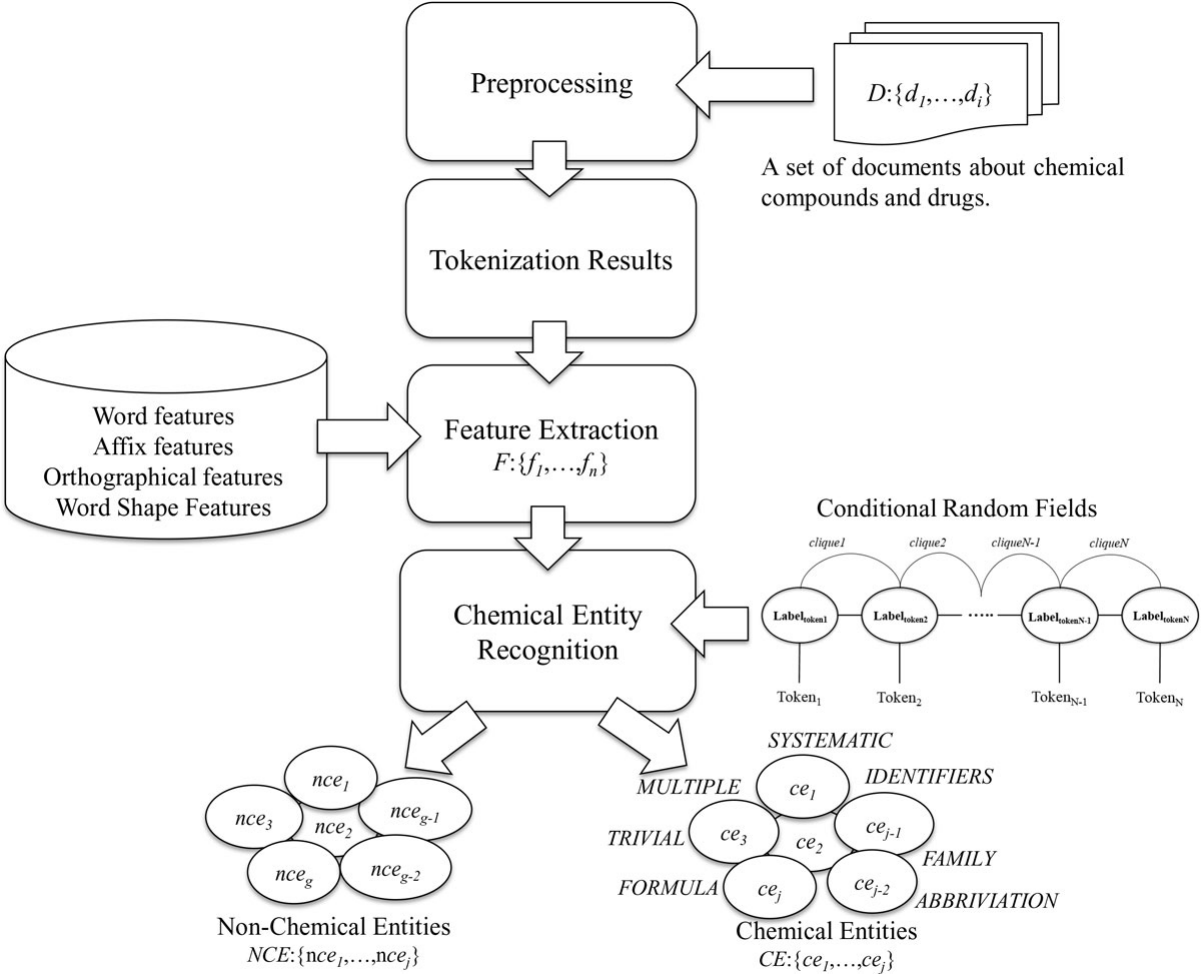
**System workflow**.

Figure 2 shows the system architecture that supports our proposed method, which comprises three key stages - *pre-processing*, *feature extraction*, and *chemical entity recognition*. First, in the pre-processing stage, a rule-based method is employed to tokenize the given document, as will be elaborated in the following subsection. After tokenization, the sentence splitter, implemented using the LingPipe package [14], is used to detect the boundary of the sentence. The GENIATagger is then utilized to generate the corresponding part-of-speech (POS) and chunk information for each token. The resulting natural language processing information, including POS and chunking, was collected by the feature extraction component to yield features. The representative text features, including word, affix, orthographical and word-shape information, are also included to enable recognition of chemical entities within a document. Finally, the chemical entity recognition stage exploits the extracted features to classify chemical entities by applying CRF. The following sub-sections elucidate each feature in detail.

### Tokenization strategy

In this study, two tokenization strategies are employed to generate different tokenization results. The performances of the generated CRF models based on these two strategies are then compared.

### Coarse-grained tokenization

In this method, the standard Penn Treebank tokenization rules [15] are utilized to tokenize the given document. The rules are summarized as follows.

• Most punctuation marks, including comma, period, and quotation markers, are separated from adjoining words.

• Contractions of verbs and Saxon genitives of nouns are split into their component morphemes. For example, "won't" becomes "wo n't".

### Fine-grained tokenization

In the fine-grained tokenization method, the coarse-grained tokenization rules are applied first. The generated tokens are then rigorously tokenized again through the following two steps:

• Add separations before and after symbols, such as hyphens and dashes.

• Separation at the locations between letters and digits, as well as at sites where a lower-case letter is followed by an uppercase letter.

### Feature sets

The feature sets that are examined in this study are based on orthographic, morphological and shallow syntactical features, which were selected mainly from our previous work proposed for the biomedical NER task [16] with a particular modification for the CHEMDNER task. These feature sets were selected because they do not rely on any specific resources and they allow researchers to re-produce and generate the comparable results. Furthermore, some of them have become the standard NER feature sets and implemented in some open-source NER systems, such as BANNER [17].

### Word features

Words that precede or follow the target word may be useful in its categorization. Consider, for example, the sentence, "**Mercury **induces the expression of cyclooxsygenase-2 and inducible **nitric oxide **synthase". If the target word is "oxide", then the following word "synthase" will help the CRF model distinguish the oxide synthase from oxide layer, enabling it correctly to classify it as a systematic-type chemical entity. In the developed model, the number of preceding/following words is set to two, and bigram and trigram words features are used as parts of the conjunction features. All of the above features were normalized to maximize the performance and to reduce the use of memory resources, as described in the authors' earlier work [16]. For example, the term "cyclooxygenase-2" was normalized to "cyclooxygenase-1" in our training set.

### Affix features

The affix of a word is a morpheme that is attached to a base morpheme to form that word. Prefixes (that precede another morpheme) and suffixes (that follow another morpheme) are two types of affix. Some prefixes and suffixes provide useful clues for classifying named entities. For example, most words that start with "hydro" are usually chemical entities with related component information. The prefixes and suffixes are defined to have between three and five characters, and they were also normalized before they were encoded into features.

### Orthographical features

The orthographical features are regular expressions that are defined to capture the style in which the names of chemical entities are written. Table 1 presents the employed features and their corresponding patterns, developed for the CHEMDNER task. Consider, for example, the FG pattern "^\d(,\d)*\-?\w+". The digits that in sequence of commas and numbers, such as '2' in the systematic name "2,4-dinitrophenyl", typlically denote the position of the attached functional groups. These patterns can be used to identify chemical entities from general English words, and are treated as binary features, whose values are unity if the sequence of tokens matches the patterns, or zero to indicate that it does not match.

**Table 1:** Orthographical features.

Feature name	Regular expression pattern
FG	^\d(,\d)*\-?\w+

INITCAPS	^[A-Z].+

CAPWORD	^[A-Z][a-z]+$

ALLCAPS	^[A-Z]+$

CAPSMIX	^[A-z]*([A-Z][a-z]|[a-z][A-Z])[A-z]*$

ALPHANUMMIX	^[A-z0-9]*([0-9][A-z]|[A-z][0-9])[A-z0-9]*$

ALPHANUM	^[A-z]+[0-9]+$

UPPERCHAR	^[A-Z]$

LOWERCHAR	^[a-z]$

SHORTNUM	^[0-9]?$

INTEGER	^-?[0-9]+$

REAL	^-?[0-9]\.[0-9]+$

ROMAN	^[IVX]+$

HASDASH	-

INITDASH	^-

ENDDASH	-$

PUNCTUATION	^[,.;:?!]$

QUOTE	^[\"`']$

### Word shape features

At times, chemical entities within the same category exhibits similar patterns, such as As(V) and DMA(V), and the word shape feature is developed accordingly. The following process is used to generate the shape of a given word: 1) all capitalized characters are replaced by "A"; 2) all non-capitalized characters are replaced by "a"; 3) all digits are replaced by "0", and 4) all non-English characters are replaced by "_". To form the second word shape feature, consecutive strings of identical characters are reduced to a single character. For instance, the term "Aaaaa_A" is contracted to "Aa_A". Consider the two chemical entities "Na(2)CO(3)" and "As(2)O(3)". The generated word shape features are "Aa_1_AA_1_" and "Aa_1_A_1_". The second word shape feature captures both chemical entities.

### Syntax features

Named entities are usually found in noun phrases, and the left or right boundaries of most chemical entities are aligned with the edge of noun phrases. For instance, in the noun phrase, "the polyhedral oligomeric silsesquioxane", the chemical name "polyhedral oligomeric silsesquioxane" aligns with the right boundary of the noun phrase. Consequently, the chunk information is encoded as a feature in our model. Moreover, POSs such as verbs and prepositions normally indicate an entity's boundary. A context window length of five is set for POS features herein.

## Results

### Dataset and evaluation metrics

The CHEMDNER text corpus [7] was utilized to examine the performance of various tag schemes. The dataset consists of 10,000 abstracts and a total of 84,355 mentions of chemical compounds and drugs that had been manually labelled by domain experts. During the BioCreative IV evaluation period, the dataset was further divided into three subsets, which were the training set (3,500 abstracts), the development set (3,500 abstracts) and the test set (3,000 abstracts). Seven categories of chemical entities adapted from the work of R Klinger, C Kolarik, J Fluck, M Hofmann-Apitius and CM Friedrich [3] were annotated in the corpus: (1) SYSTEMATIC: the systematic names, such as IUPAC; (2) IDENTIFIERS: database IDs, including CAS numbers, PubChem IDs, company registry numbers, ChEBI and CHEMBL IDs; (3) FORMULA: molecular formula, SMILES, InChI, or InChIKey; (4) TRIVAL: trivial, brand, common or generic names of compounds; (5) FAMILY: chemical families that can be associated to chemical structures; (6) MULTIPLE: mentions that correspond to chemicals that are not described by a continuous string of characters; (7) ABBREVIATION: abbreviations and acronyms.

In this study, the procedure for executing the BioCreative IV CHEMDNER task is utilized to report the system performance in two tasks: the indexing of chemical documents (CDI) and the recognition of mentions of chemical entities (CEM). Given a set of documents, the CDI system returns a list of unique chemical entities for each, whereas the CEM system provides exact occurrence information for all mentioned chemical entities. For both tasks, the official evaluation script that is released by BioCreative IV is used to determine the performance in terms of the micro-average recall (R), precision (P) and the balanced F-measure (F).

$${\text{R}}_{micro}=\frac{\text{TP}}{\text{TP}+\text{FN}}\text{,}{\text{P}}_{micro}=\frac{\text{TP}}{\text{TP}+\text{FP}}\text{,}{\text{F}}_{1}=\frac{2\times \text{Recall}\times \text{Precision}}{\text{Recall}+\text{Precision}}$$

True positive (TP) refers to the number of correctly recognized chemical mentions. False negative (FN) is the number of human-annotated chemical mentions that were omitted by the presented system. False positive (FP) is the number of recognized chemical mentions that were not annotated by human annotators. The result shows the overall system performance independent of the categories of the chemical entities.

With respect to the CEM task, the category-pivoted measure is evaluated by considering the individual RPF-scores of the seven chemical entity categories, and the macro-average RPF-scores. Since the original evaluation script does not yield the category-pivoted results, the original gold annotations are split into seven sub-annotation files with regard to their category *C*, and then calculated the RPF-score for each category using the official evaluation script. Finally, the following equations were utilized to report the category result in CEM.

$${\text{R}}_{macro}=\frac{1}{\left|C\right|}\sum_{i=1}^{\left|C\right|}\frac{{\text{TP}}_{i}}{{\text{TP}}_{i}+{\text{FN}}_{i}},{\text{P}}_{macro}=\frac{1}{\left|C\right|}\sum_{i=1}^{\left|C\right|}\frac{{\text{TP}}_{i}}{{\text{TP}}_{i}+{\text{FP}}_{i}}$$

### Experimental configurations

The four tag schemes that were described in the Methods section determine the four configurations in our experiments-IOB, IOBE, IOBES and IOB_12_E-all of which use the feature sets that were described in the Methods section. Two tokenization strategies were adopted to investigate the impact of the tokenization method on each tag scheme. The first directly exploits the tokenization results that are generated by following the coarse-grained tokenization rules, and the second uses the rules that were described in the Fine-grained tokenization sub-section to produce tokens with a finer granularity. The subscripts "f" in the following notations is used to distinguish the configurations with fine-grained tokenization from the first configurations, IOB_f_, IOBE_f_, IOBES_f _and IOB_12_E_f_. For the CEM task, a fixed confidence of 0.5 was empirically set for all configurations in selecting the recognized chemical entities. The CDI results were converted from the CEM results by removing all duplicate entities and sorting them in order of descending confidence.

### Results for CDI and CEM

First, the performance of different tag sets on the development set was studied. Table 2 presents the results obtained when the four tag schemes were combined with the tokenization results that were derived by using the coarse-grained tokenization rules. Two tag schemes performed better than the others in the CDI and CEM tasks; the IOB scheme yielded the best F-score in the CDI task, while the IOBES scheme obtained the best F-score in the CEM task. Overall, the IOB scheme provided the best precision in both tasks, while the IOBES scheme resulted in the best recall. The most representative tag set, IOB_12_E, yielded the worst overall RPF-scores of the four configurations.

**Table 2:** The CDI and CEM results on the CHEMDNER development set.

Config	CDI			CEM		
	
	P (%)	R (%)	F (%)	P (%)	R (%)	F (%)
IOB	**78.40**	74.60	**76.45**	**80.37**	68.74	74.10

IOBE	76.53	75.53	76.03	78.78	69.61	73.91

IOBES	76.57	**75.98**	76.27	78.88	**70.10**	**74.23**

IOB_12_E	50.70	56.48	53.43	54.28	55.51	54.88

The fine-grained tokenization method is paired up with the four configurations, and the performance achieved in each case is displayed in Table 3. Unlike in Table 2 when finer tokenization is employed, the IOBES_f _scheme outperforms the other three tag schemes as measured by all of the RPF-scores for both tasks. Furthermore, in order to confirm whether the slightly better performance between the IOBES_f _and the other two less representative tag schemes (IOB_f _and IOBE_f_) is statistically significant, a two-sample paired t-test is applied. The null hypothesis states that there is no difference between the two configurations. To retrieve the average F-scores and their deviations required for the t-test, we merged the training set and development set and randomly sampled thirty new training sets and thirty new development sets according to the size of the original training/development sets. Afterwards, we summed the scores for all thirty development sets and calculated the averages for performance comparison. The results reveal that IOBES_f _outperforms IOB_f_, IOBE_f _and IOB12E_f _with statistical significance. While more precise and expressive tag schemes seems to perform better in combination with finer tokenization, the most representative IOB_12_E_f _remains the worst of all schemes.

**Table 3:** The CDI and CEM results on the CHEMDNER development set with the finer tokenization.

Config	CDI			CEM		
	
	P (%)	R (%)	F (%)	P (%)	R (%)	F (%)
IOB_f_	83.24	81.01	82.11	83.68	77.25	80.33

IOBE_f_	83.37	81.13	82.23	83.71	77.31	80.38

IOBES_f_	**83.96**	**81.57**	**82.75**	**84.08**	**77.87**	**80.85**

IOB_12_E_f_	54.31	56.14	55.21	57.17	58.88	58.01

**Figure 3:**
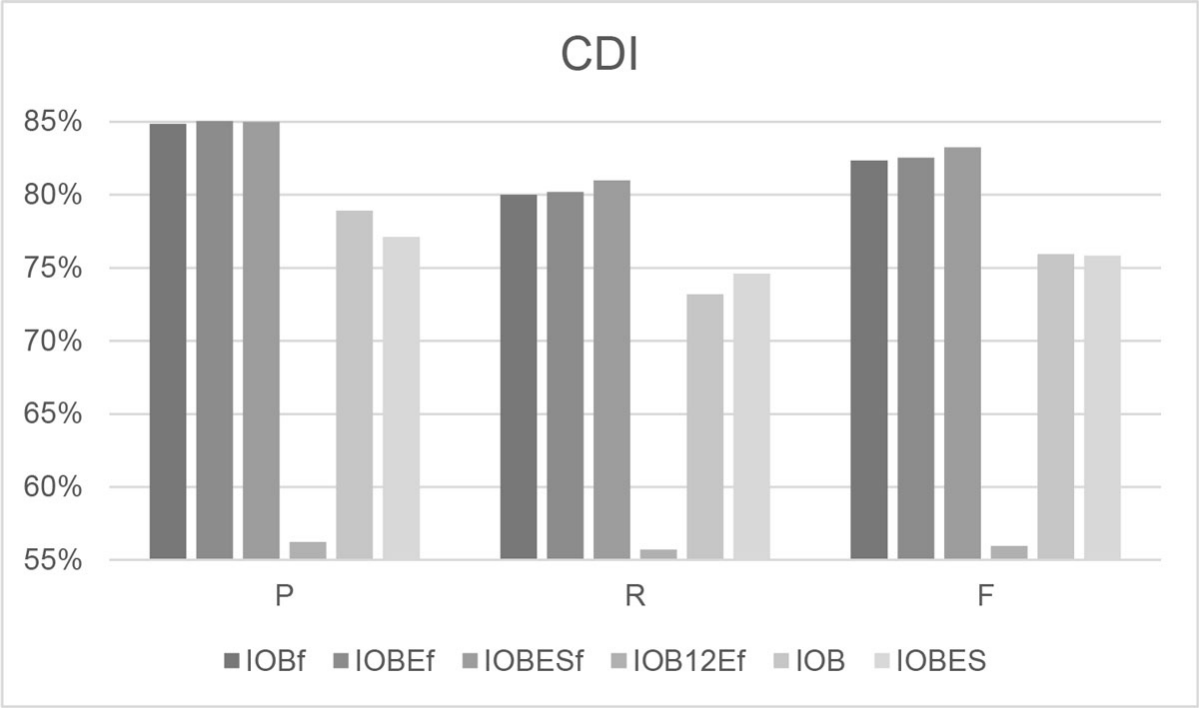
**The CDI results on the CHEMDNER test set**.

**Figure 4:**
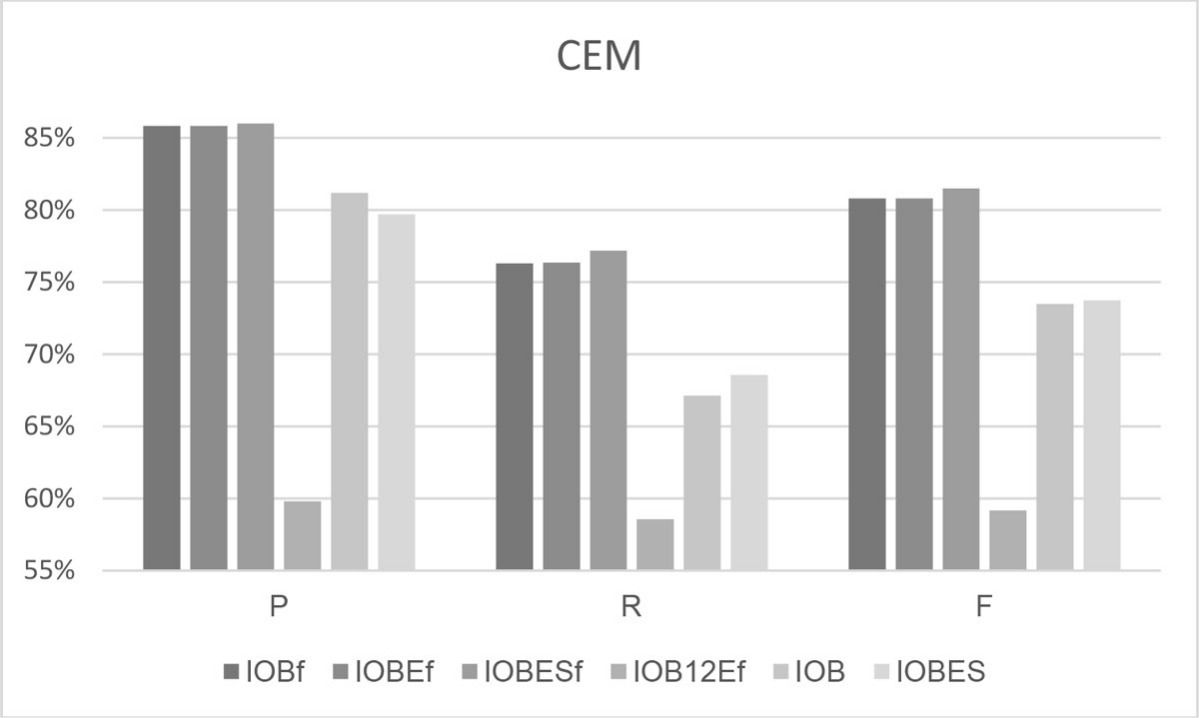
**The CEM results on the CHEMDNER test set**.

Figures 3 and 4 compare the performances of the four fine-grained tokenization-based tag schemes, with those of the IOB and IOBES schemes, when applied to the test set of the CDI and CEM tasks. The graphs reveal that the IOBES_f _configuration provided the best RPF-scores of all six configurations, as it achieved F-scores of 0.833 and 0.815 in the CDI and CEM tasks, respectively, and so outperformed the most-adopted tag sets, IOB and IOB_f_, in terms of F-scores by 0.08 and 0.01 in both tasks. Similar to the results of the development set, the IOBES performs slightly better than IOB in the CEM task, while the IOB performs better in the CDI task. The IOB_12_E_f _remains to exhibit the worst performance of all schemes.

### The category results of CEM

Table 4 displays the results concerning categories of chemical mentions on the test set. Owing to the limited space, Figure 4 presents only the best five configurations shown in Figure 3, and the best RPF-scores for each category are presented in bold. The overall results (last row) shows that the IOBES_f _outperformed the other configurations in terms of all three macro-average RPF scores, and had the best R and F-scores in all categories except the Multiple category.

**Table 4:** The CEM category results on the CHEMDNER test set.

	IOBES			IOBES_f_			IOBE_f_			IOB			IOB_f_		
	
	P	R	F	P	R	F	P	R	F	P	R	F	P	R	F
Abbr.	84.1	56.7	67.8	**87.5**	**59.6**	**70.9**	87.5	59.2	70.6	82,9	47.1	60.1	87.2	59.1	70.5

Fam.	82.5	64.6	72.4	**85.1**	**70.0**	**76.8**	85.0	69.3	76.2	82.5	63.5	71.8	84.3	68.9	75.8

Form.	79.0	62.9	70.0	**83.1**	**69.0**	**75.4**	83.0	68.8	75.2	74.4	58.3	65.4	83.0	68.4	75.0

Ident.	91.2	46.4	61.5	94.0	**70.8**	**80.8**	93.9	69.6	80.0	93.6	70.8	80.6	**94.7**	70.2	80.6

Mult.	46.3	38.2	41.9	77.0	38.7	51.5	**83.0**	**39.2**	**53.2**	78.8	31.7	45.2	75.6	34.2	47.1

Sys.	82.4	61.2	70.2	85.2	**81.3**	**83.2**	**85.4**	80.1	82.6	78.7	70.2	74.2	85.2	79.9	82.5

Tri.	86.4	78.6	82.3	**89.9**	**81.5**	**85.5**	89.1	81.2	85.0	85.6	73.0	78.8	89.0	81.2	84.9

All	76.8	66.0	71.0	**82.7**	**73.9**	**78.1**	82.5	73.4	77.6	77.9	64.4	70.5	82.3	73.2	77.5

In summary, the widely adopted IOB tag scheme provides generally a good formulation of the word sequences used in chemical mentions, because it can achieve comparable RPF-scores to more representative tag schemes with less usage in time and memory during the training phase. For instance, using the IOB tag in comparison to IOBES, both the memory demand and training time are around three time less. When coarse-grained tokenization rules are used, more representative tag scheme exhibited no significant advantage over the IOB tag scheme. Nonetheless, as pointed out by M Krallinger, F Leitner, O Rabal, M Vazquez, J Oyarzabal and A Valencia [7], the development of a set of specialized tokenization rules for the recognition of chemical terms is required. The experimental results herein support this assertion, and revealed a significant improvement of approximately 6.23% in the F-score between IOB and IOB_f _with an increased memory usage (6.5%) and training time (23.3%). Our discovery indicates that under the finer tokenization strategy, the more representative IOBES tag set should be preferred over others for performing the CHEMDNER task. Given the higher training cost in the three time of time and memory usages, we can anticipate a further boost of the RPF scores and a stable improvement in the recognition of all chemical entity categories in both the CDI and CEM tasks.

## Discussion

The experiments herein were carried out to investigate the influence of tokenization strategy and effective tag scheme on the CHEMDNER task. Comparing the predicted boundaries of the chemical mentions obtained using the best configuration, IOBES_f_, in the experiments with those obtained using its counterpart, IOBES, and the most adopted tag set, IOB_f_, revealed that the use of fine-grained tokenization precisely identifies any modification of arbitrary symbols on chemicals and clearly defined their boundaries. For instance, with fine-grained tokenization, the system can retrieve "octadecanol" rather than "octadecanol-covered" from the sentence that includes "...with an octadecanol-covered Au(111) surface investigated...". Likewise, from the sentence that includes "Among the artemisinin-based combination therapy (ACT) regimens...", "artemisinin" rather than "artemisinin-based" is recognized. The tokenization strategy also enables the CRF model to recognize entities mentioned that are mentioned next to forward slashes, such as those in "alcohols/esters", "Plu/PAA/Epi" and "His/Tyr", which are often used in descriptions of a group of chemicals with similar attributes.

Furthermore, the generation of fine-grained tokens along with the more representative tag set allows the CRF model to capture better mentions of longer entities that were generated by the fine-grained tokenization method. For example, in coarse-grained tokenization, two and four tokens were generated for "N-cinnamoylated chloroquine" and "10, 12-pentacosadiynoic acid", respectively. Either were overlooked in the coarse-grained tokenization method, or have incomplete boundaries in tag schemes such as IOB. The IOBES tag scheme with fine-grained tokenization can successfully recognize entities that comprise many tokens. For instance, the IOBES_f _model can correctly recognize the boundary of the chemical entity "α-phenyl-N-tert-butyl nitrone" using ten tokens after fine-grained tokenization.

Since the purpose of the CHEMDNER task is to recognize a sequence of words with various lengths that specify a chemical entity, the distribution of entity name lengths in the corpus after tokenization serves as an essential factor in determining the selection of tag scheme. Table 5 presents the distribution of lengths of the name of chemical entities in the CHEMDNER dataset following the fine-grain tokenization.

**Table 5:** The distribution of chemical entities with different lengths in the CHEMDNER corpus.

Entity Length	Training (%)	Development (%)	Test (%)	Overall (%)
1	70.50	70.82	71.45	70.90

2	9.33	9.29	8.99	9.21

3	6.28	6.12	6.28	6.22

4	4.63	4.43	4.06	4.39

≦4	90.73	90.66	90.79	90.72

4 <	9.27	0.34	9.21	9.28

According to our hypothesis, the use of tag schemes, which can capture words that comprise a chemical entity using different tags that identify their relative positions within the name of the entity, should enhance the preciseness of entity recognition. As a result, the utilization of a more explicit tag set, such as IOBE, increased the accuracy of identification of chemical entities with longer names. In the sentence "Novel **N-indolylmethyl substituted spiroindoline-3,2'-quinazolines **were designed as potential inhibitors of SIRT1", the tag set IOBE can retrieve the correct chemical name in bold, whereas IOB recognized "N-indolylmethyl" and "spiroindoline-3,2'-quinazolines", respectively. Similarly, IOBE recognized the name "N-cinnamoylated chloroquine" in the expression "**N-cinnamoylated chloroquine **analogues as dual-stage antimalarial leads", whereas IOB determined two names "N-cinnamoylated" and "chloroquine". Specification of the end of a chemical name within the IOBE set rather than simply regarding it as a part of the name seems to improve the recognition of chemical entities with long names.

Since words that consist of four or fewer tokens have constitute around 90.7% of the CHEMDNER corpus, the five-tag scheme, IOB_12_E, should outperform the four tag scheme IOBE. However, increasing the rigidity of the tag set does not provide any improvement, as revealed by the fact that the IOB_12_E performed most poorly. Close scrutiny reveals that the use of the IOB_12_E tag set makes difficult the recognition of chemical names that consist of two words, such as "ammonium sulphate", "allyl alcohol" and "acetate esters", which are 9.2% of the names in the CHEMDNER corpus. Since IOB_12_E captures not only the first word (B_1_), but also the word that follows it (B_2_), it may have trouble with the recognition of two-token entities, in which the second word serves both as B_2 _and the end, or is an independent entity itself. For names with a single word, which occupies 70.9% of the entities in the CHEMDNER corpus, the addition of the S tag to form the IOBES tag scheme provides an improvement over IOBE and IOB. Therefore, we believe that the IOBES tag scheme with fine-grained tokenization is the best alternative for capturing sufficient discriminative information for the CHEMDNER task.

### Comparison with the other CHEMDNER systems in BioCreative IV

Table 6 compares the performance of the best tag scheme (IOBES*_f_*) with those of the most highly ranked CRF-based systems when applied to the CHEMDNER test set. Notably, the corresponding results cannot be directly compared because these systems are all based on different feature sets and lexicon resources. For example, R Leaman, C-H Wei and Z Lu [11], the top ranked system, proposed the use of semantic binary features to represent chemical characteristics, such as alkane stems (such as "meth," "eth," and "tetracos"), and trivial rings (such as "benzene," and "pyridine"). They also used several patterns and the ChemSpot system [18] to capture potential mentions of chemicals and encoded the outputs as features. Their system, System 1, was the top-ranked system. System 2, developed by Y Lu, × Yao, × Wei and D Ji [19], used a word clustering algorithm to group words with similar meanings and encoded the semantic groups as features for CRF training. RT Batista-Navarro, R Rak and S Ananiadou [10] (System 3) represented several lexicon resources, such as ChEBI, and DrugBank, as dictionary features. However, this study uses only the basic feature sets, which have been confirmed by most studies to perform well in biomedical NER tasks [20]. Furthermore, the aforementioned systems all used different pre-/post-processing steps to fine-tune the recognition performance. For instance, all systems used different tokenization mechanisms and abbreviation recognition tools to improve the recall rate.

**Table 6:** Comparison the results with the top 3 CEM systems in the CHEMDNER test set.

System	P (%)	R (%)	F (%)
System1: Leaman *et al*.	89.09	85.75	87.39

System2: Lu *et al*.	89.10	85.20	87.11

System3: Batista-Navarro *et al*.	92.67	81.24	86.58

IOBES*_f_*	86.32	77.18	81.49

Average	81.59	72.54	78.59

Average refers to the average score of all participating teams in the CEM task. The IOBES*_f _*configuration is ranked tenth out of 26 participating teams in the CHEMDNER task.

Whereas comparing the aforementioned advanced features and pre-/post-processing are beyond the scope of this work, the observations herein may be useful for further improving the aforementioned systems. As demonstrated experimentally, the five-tag scheme IOBES outperformed all others. Therefore, we believe that the performance of Systems 1 and 3 can be further improved since both adopted the simplest tag scheme, IOB. System 2 is the only system that utilized the IOBES tag scheme in the CHEMDNER task. However, System 2 did not pay attention to the effect of the tokenization in the combination with tag scheme. As established by P Corbett, C Batchelor and S Teufel [21] and S Eltyeb and N Salim [20], tokenization is an important issue in CHEMDNER systems, and a customized tokenizer can provide clear advantages in the handling of multi-token chemical entities. Therefore, a good CHEMDNER system must have a specialized tokenizer or be effective in handling multi-token names. This study demonstrated that properly choosing the representative tag scheme to be used with the fine-grained tokenization strategy, can better capture multi-token words in a chemical name. We therefore believe that the aforementioned systems can be improved by adopting to them with the proposed fine-grained tokenization strategy.

## Conclusions

This study describes a system that is developed for performing the CHEMDNER task, and it specifically examined the effect of tokenization and different representative tag sets on chemical and drug name recognition. The use of finer tokenization was generally associated with better performance of all tag sets. Moreover, of all the tag sets used, delicate tag schema such as the five-tag scheme IOBES provided better performance than the others. However, the complexity of the tag set is not entirely correlated with the proficiency of CHEMDNER, as the results herein revealed that the IOB_12_E tag set performed the worst overall. In summary, finer tokenization combined with the elaborate tag set IOBES achieved the best performance in recognizing chemical and drug names.

## Competing interests

The authors declare that they have no competing interests.

## Declarations

The research was financially supported by the Ministry of Science and Technology Republic of China, Taiwan, under Contract No. MOST-103-2221-E-038-019.

This article has been published as part of *Journal of Cheminformatics *Volume 7 Supplement 1, 2015: Text mining for chemistry and the CHEMDNER track. The full contents of the supplement are available online at http://www.jcheminf.com/supplements/7/S1.
